# Adipokine Levels and Glucose Metabolism Responses to the Acute Effects of High‐Intensity Interval Exercise (HIIE) and Moderate Intensity Continuous Exercise (MICE) in Prediabetic Obese Men

**DOI:** 10.1002/edm2.70125

**Published:** 2025-11-09

**Authors:** Hadi Golpasandi, Mohammad Rahman Rahimi, Elham Elhami, Mahan Moludi

**Affiliations:** ^1^ Department of Exercise Physiology University of Kurdistan Sanandaj Iran

**Keywords:** adiponectin, GDF‐15, glucose metabolism, HIIE, MICE, prediabetes

## Abstract

**Introduction:**

Prediabetes involves impaired glucose regulation and alterations in adipokines. The aim of this study was to investigate the immediate effects of high‐intensity interval training (HIIE) and moderate‐intensity continuous exercise (MICE) on metabolic function in obese prediabetic individuals.

**Methods:**

Eight obese prediabetic men (30–45 years) were assigned to three conditions in a crossover design: HIIE, MICE and control. The HIIE protocol consisted of 10 × 1 min bursts at 90%–100% HRmax with active rest, and the MICE protocol consisted of 30 min of continuous exercise at 60%–65% HRmax. Blood samples were collected at four time points (before, immediately, 3 and 24 h after exercise) and serum levels of GDF‐15, adiponectin, leptin and OGTT markers were measured.

**Results:**

Results of repeated‐measures ANOVA showed that the effect of time on leptin levels was significant (*p* < 0.05), and both types of exercise resulted in significant decreases compared to the control group (*p* < 0.05), although no difference was observed between HIIE and MICE (*p* > 0.05). Serum adiponectin levels increased significantly after MICE compared with baseline and control groups (*p* < 0.05), whereas no significant changes were observed in HIIE; however, there was no significant difference between the two exercise conditions (*p* > 0.05). GDF‐15 levels increased significantly after both HIIE and MICE training, with no significant difference between the two exercise conditions (*p* > 0.05). Both HIIE and MICE differently altered the glucose response pattern over time during the OGTT, but the average total glucose did not differ significantly between the two exercise groups (*p* > 0.05).

**Conclusion:**

HIIE and MICE exercises reduced leptin and altered glucose response patterns (at 90 min) in obese prediabetic subjects, without significant differences between them. The type and intensity of exercise regulate metabolism, but more studies are needed.

## Graphical abstract

In a cross‐sectional study (*N* = 8 obese prediabetic men), high‐intensity interval exercise (HIIE) and moderate‐intensity continuous exercise (MICE) were compared with a control group. Both exercises significantly reduced serum leptin and increased GDF‐15 levels (*p* < 0.05), and MICE uniquely increased adiponectin (*p* < 0.05). The glucose response during the OGTT at 90 min after exercise was altered in both conditions, with no difference between HIIE and MICE. These findings highlight the exercise‐induced modulation of adipokines and glucose metabolism in prediabetes.

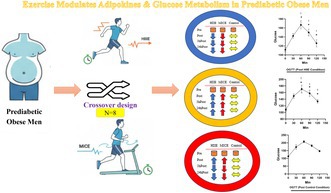

## 1 Introduction

The global epidemic of obesity and prediabetes, as two important metabolic disorders, has caused widespread concern in the field of public health [1]. Prediabetes, an intermediate stage between normal glucose levels and higher‐than‐normal levels in type 2 diabetes, is associated with insulin resistance, impaired lipid metabolism, and an increased risk of cardiovascular disease [2]. One of the hallmarks of obesity and prediabetes is the imbalance in the secretion of adipokines from adipose tissue, which can play a pivotal role in the development and progression of metabolic disorders [3]. Adipokines such as adiponectin and leptin are among the most important factors influencing the regulation of insulin sensitivity, glucose homeostasis and energy balance. In particular, decreased adiponectin and increased leptin, also known as leptin resistance, have been frequently reported in obese and prediabetic patients [4].

In the meantime, growth differentiation factor 15 (GDF‐15) has attracted the attention of researchers in recent years as one of the emerging adipokines that is mainly secreted in response to metabolic, inflammatory and mitochondrial stresses. GDF‐15 is normally expressed at low levels in various tissues, but it increases significantly under conditions of injury, inflammation or impaired metabolism [5]. In addition to having a protective role in many organs, this protein can act as a metabolic sensor and play a role in regulating appetite, body weight, glucose tolerance and the activity of other adipokines such as adiponectin and leptin [6, 7].

Experimental and human studies suggest that GDF‐15 may interact with signalling pathways related to AMPK, PGC‐1α and other metabolic pathways, and interact with adipokines in regulating metabolism [8, 9]. There are also findings that show that GDF‐15 increases in response to physical activity, but the pattern of its response is highly dependent on the type, intensity and duration of exercise, as well as the individual's metabolic status [10, 11, 12]. For example, in studies conducted on individuals with coronary heart disease, an acute increase in GDF‐15 levels has been reported after endurance exercise, while the response may be different in obese or diabetic individuals [13]. Given that prediabetes is an intermediate state with less chronic inflammation and metabolic stress compared to overt diabetes, individuals with prediabetes may still retain a greater degree of metabolic flexibility. Therefore, one would expect that the response of GDF‐15 to acute exercise—particularly high‐intensity interval exercise (HIIE), which imposes a higher metabolic challenge—would differ between individuals with prediabetes and individuals with obesity or type 2 diabetes. Examining this distinction could help clarify the role of GDF‐15 in early versus advanced stages of metabolic disorders.

In this context, exercise training is recognised as one of the most effective non‐pharmacological interventions for improving insulin sensitivity and controlling glucose metabolism [14]. In particular, high‐intensity interval training (HIIT) and moderate‐intensity continuous training (MICT) have diverse effects on metabolic indices due to their different physiological mechanisms [15]. HIIT training, by creating higher metabolic and hormonal stress, can induce stronger inflammatory and adipokine responses and lead to greater improvements in glucose tolerance and body composition, while MICT training shows its effects by creating more lasting adaptations, especially in adipose tissue and the cardiovascular system [16]. However, the available information on acute adipokine responses to different types of exercise, particularly in the context of GDF‐15, remains sparse and limited. In a study by Kleinert et al. (2018), a significant increase in serum GDF‐15 levels was observed after a single session of high‐intensity cycling [10], while in another study, a significant increase in GDF‐15 was reported after 12 weeks of aerobic exercise in obese subjects [17], which was not similar in subjects with type 2 diabetes [18, 19]. Also, a significant decrease in serum GDF‐15 levels was reported 24 h after acute HIIE and MICE exercise bouts in obese men, which was accompanied by a significant decrease in serum glucose levels [11]. In other studies, it was shown that a long‐term period of exercise training at different intensities significantly reduced GDF‐15 levels in prediabetic individuals [18, 20].

Regarding the relationship between GDF‐15 and adiponectin, it was recently reported that a high GDF‐15 to adiponectin ratio was independently associated with an increased likelihood of type 2 diabetes in all study groups, and this ratio could be a better indicator of type 2 diabetes [6]. Furthermore, the relationship between changes in GDF‐15 and other adipokines such as leptin, especially in response to acute exercise sessions, has been less studied.

Most previous studies have focused on chronic exercise responses, and few data are available on acute and immediate changes in adipokines and glucose tolerance indices after a single exercise session, especially in obese prediabetic men. However, examining acute responses can provide detailed insight into the primary mechanisms of exercise effects and contribute to the design of more effective exercise interventions for high‐risk populations. Therefore, given the lack of research in this field and the lack of a study that has examined and compared the acute responses of HIIT and MICT exercises on GDF‐15 levels and its relationship with adiponectin, leptin, serum glucose and glucose tolerance index in a population of obese prediabetic men, the present study aims to fill this gap and provide a better understanding of the potential role of GDF‐15 in regulating glucose metabolism through the adipokine axis by closely examining these indices after a single exercise session.

## 2 Materials and Methods

### 2.1 Population and Sample

The statistical population of this study consisted of obese men with prediabetes who were recruited through a public call at health centers and urban cyberspace. After an initial screening, 10 eligible subjects were purposively selected from among the volunteers. The participants in this study were obese men with prediabetes who were selected based on specific criteria. Inclusion criteria included age between 30 and 45 years, body mass index (BMI) between 30 and 35 kg/m^2^, diagnosis of prediabetes based on one of the fasting plasma glucoses (FPG) indices between 100 and 125 mg/dL, or 2 h after an oral glucose tolerance test (OGTT) between 140 and 199 mg/dL, or HbA1c within the range of 7.5 to 4.6% according to the American Diabetes Association (ADA) guidelines, and not taking metformin. Volunteers should not have had a history of taking blood sugar‐lowering drugs, especially metformin, in the last 3 months, as evidence suggests that metformin can independently increase the level of growth differentiation factor 15 (GDF‐15) and act as an interfering factor in the analysis of the results. Also, volunteers should not have participated in regular exercise programs in the last 3 months, have no history of cardiovascular, renal, hepatic diseases, type 2 diabetes, or hormonal disorders, have not used nutritional supplements or drugs that affect metabolism, and have refrained from smoking or alcohol consumption. Finally, the ability to fully cooperate in the research stages and sign an informed consent form were other requirements for inclusion in the study. Exclusion criteria also included the occurrence of any injury, failure to cooperate in the implementation of the protocol, or voluntary withdrawal.

### 2.2 Determining Sample Size and Study Design

The sample size was calculated using GPower software and according to previous results in similar studies, considering a statistical power of 80%, a significance level of 0.05 and a medium effect size, and was determined as 10 people. This study was conducted as a cross‐over quasi‐experimental design. Each subject participated in three experimental conditions: (1) high‐intensity intermittent exercise (HIIE), (2) moderate‐intensity continuous exercise (MICE) and (3) a control condition without activity. The order of participation in different conditions was randomised (Randomised Latin Square Design) and a one‐week washout period was observed between each session to eliminate lasting effects.

Based on a priori power analysis, 10 participants were needed; however, two withdrew before the start of the exercise protocol, and ultimately 8 participants completed the study.

### 2.3 Initial Orientation and Assessment Session and Graded Exercise Test (GXT)

Before the intervention, a meeting was held to familiarise participants with the objectives, research implementation and measurement methods. A medical history and disease history questionnaire was completed by eligible individuals. Then, anthropometric characteristics including weight, height, body mass index (BMI) and body fat percentage (using a BIA device, InBody 270 model, South Korea) were measured and recorded.

### 2.4 GXT Test

In this study, participants performed a test (GXT) on a stationary bicycle (Lode Excalibur Sport) to measure maximum heart rate (HR_max_) and maximum power output (W_max_). The GXT protocol consisted of 3 min of cycling at 60 rpm and 50 watts, which was increased by 25 watts every 3 min for the first three stages and then increased every 1 min until participants could no longer maintain 50 rpm. Maximum heart rate (HR_max_) was derived from the highest heart rate recorded by the 12‐lead electrocardiograph system during the test [21]. Maximal power output (W_max_) was used to calculate workload in the HIIE or MICE interventions in the main trial. Perceived intensity of exertion (RPE) was recorded using the Borg scale of 6–20. One to three weeks after the screening session, participants were randomly assigned to the HIIE or MICE intervention groups, based on gender and BMI. The GXT protocol in the present study was modified from Parker et al., (2017).

### 2.5 Exercise Protocols HIIE and MICE

**FIGURE 1:**
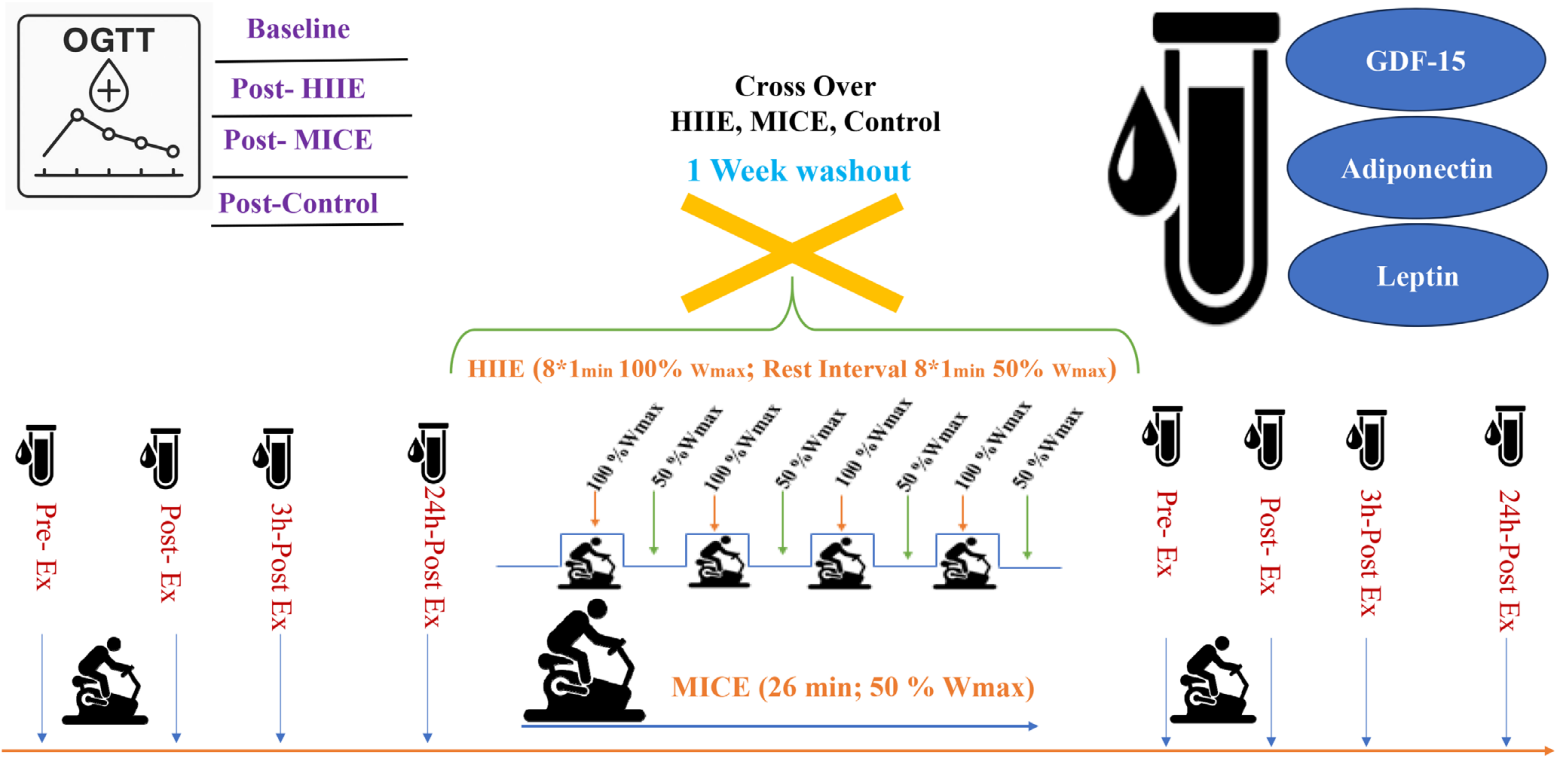
Schematic diagram of the exercise intervention protocol in obese prediabetic men.

In the HIIE condition, subjects performed a 1:1 activity‐rest pattern designed based on a study (Parker and et al., 2017), including a 5‐min warm‐up at 50% of the participants' W_max_ achieved during GXT. After the warm‐up, participants performed 8 × 1‐min cycling bouts at 100% W_max_, interspersed with a 1‐min active recovery bout of cycling at 50 W. This was followed by a 3‐min cool‐down at 50% W_max_. The total duration of the exercise session was 24 min. The MICE session consisted of 38 ± 1 min cycling at 50% of the participants' W_max_ [21]. In the control condition, the subjects were in a completely relaxed, sitting position in the laboratory environment and did not perform any exercise, (Table 1 and Figure 1).

**TABLE 1:** Summary of high‐intensity interval exercise (HIIE) and moderate‐intensity continuous exercise (MICE) protocols after work matching, and the control condition.

Protocol	Phase	Duration (min)	Intensity/power
HIIE	Warm‐up	5	50% W_max_
	Intervals + recovery	16	8 × 1 min 100% W_max_ + 8 × 1 min 50 W
	Cool‐down	3	50% W_max_
Total duration HIIE	—	24	—
MICE (work‐matched)	Continuous cycling	26.7 ± 1	50% W_max_
Control	Rest	26–38	—

To equalise the protocols, the total mechanical work of the HIIE session (sum of power × time including warm‐up, intervals, active recovery and cool‐down) was calculated for each participant. The duration of the MICE session was then adjusted to equalise the total mechanical work of the HIIE. For example, with an average W_max_ of 300 W, the MICE duration was set to approximately 26.7 min at 50% W_max_. This approach allowed both protocols to be identical in terms of training volume while maintaining the intensity and training pattern characteristics inherent to each modality. This design allows for direct comparison of the physiological responses elicited by the training modality (Table 1 and Figure 1).

### 2.6 Blood Sampling

Blood samples were collected from all participants at four times: before exercise, immediately after exercise, 3 and 24 h after exercise under fasting conditions (at least 10 h) from the antecubital vein using vacuum tubes. After centrifugation of the samples at 3000 rpm for 10 min, serum was separated and stored at −80°C until analysis (Figure 1).

### 2.7 Measurement of Adipokines

To measure serum levels of adipokines GDF‐15, adiponectin and leptin, ELISA kits manufactured by antibodies‐online, Germany, catalogue GDF15: ABIN7274723, sensitivity: 6.5 pg/mL; Adiponectin: ABIN6953508, sensitivity: 0.069 ng/mL; Leptin: ABIN6574137, sensitivity: 0.054 ng/mL; were used. All experiments were performed in duplicate according to the manufacturer's instructions. Results were reported in picograms per millilitre or nanograms per millilitre.

### 2.8 Statistical Method

Descriptive indicators including mean and standard deviation, percent change in mean and 95% confidence interval were used in data analysis. The Shapiro–Wilk test was used to examine the normality of data distribution. If the distribution was normal, repeated measures ANOVA was used to compare variables in different time and intervention conditions. If a significant difference was observed, the Bonferroni post hoc test was used for pairwise comparisons. The significance level in all tests was considered to be 0.05, and all statistical analyses were performed using GraphPad Prism software version 9.5.

## 3 Results

The physiological and anthropometric characteristics of the subjects are presented in Table 2.

**TABLE 2:** Physiological and anthropometric characteristics of the study participants.

Variable	Age (years)	Weight (kg)	Height (cm)	BMI (kg/h^2^)	HR_rest_ base	BP_rest_ base
Subjects	38.9 ± 5.16	92.8 ± 8.5	174.1 ± 5.3	31.6 ± 1.8	77.2 ± 5.9	130.6 ± 7.8

*Note:* (*n* = 8).

### 3.1 The Effects of Acute HIIE and Continuous Moderate‐Intensity MICE Exercise on GDF‐15 Levels

**FIGURE 2:**
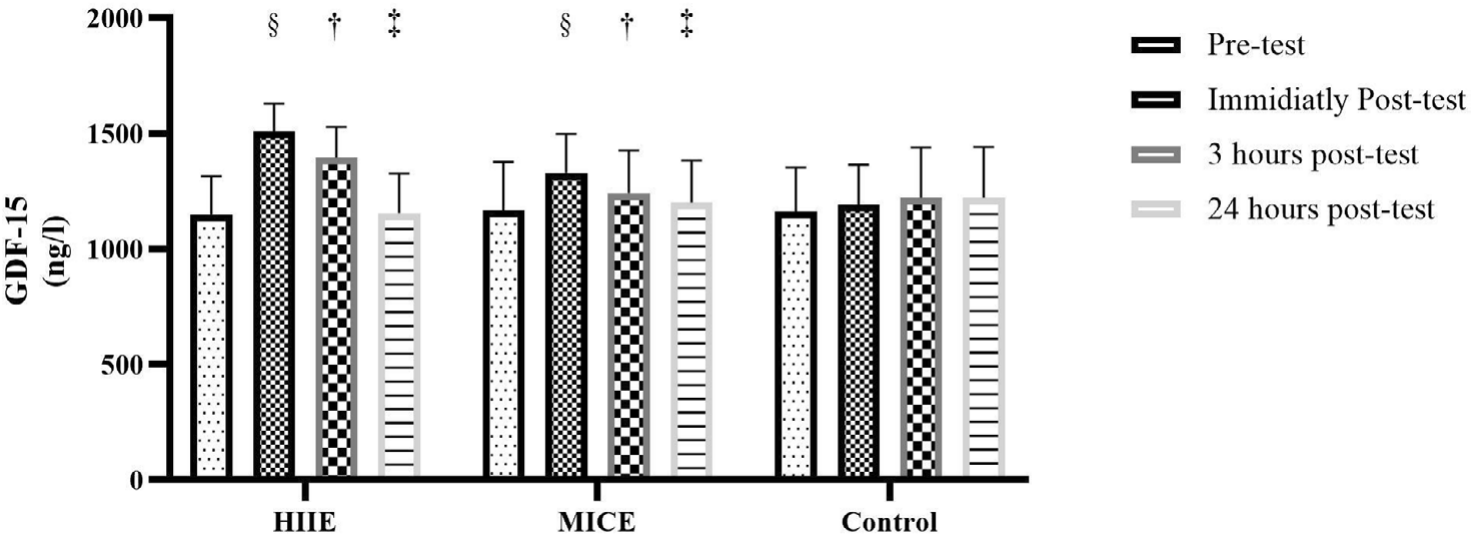
Acute effects of high‐intensity interval exercise (HIIE) and moderate‐intensity continuous exercise (MICE) on GDF‐15 levels. (§) Significant difference compared with pre‐test (baseline). (†) Significant difference compared with immediately post‐test. (‡) Significant difference compared with 3 h post‐test.

The effect of time on serum GDF‐15 levels was examined using a repeated measures ANOVA. The results of the intra‐individual test, with Greenhouse–Geisser correction, indicated that there was a significant difference in GDF‐15 levels at the four measurement times (*F* (2.16, 45.33) = 170.10, *p* < 0.001). The effect size of these changes was reported to be very high (Partial Eta Squared = 0.890), indicating a high strength of the effect of time. The statistical power of this test was also 1.000, indicating a very high confidence in detecting real time differences (Figure 2).

On the other hand, the between‐group effect due to different training conditions (HIIE, MICE and control) was not significant (*F* (2, 21) = 0.695, *p* = 0.510). This result indicates that overall, GDF‐15 levels did not differ significantly independent of the type of training intervention. However, the effect size in this analysis was small (Partial Eta Squared = 0.062) and the low statistical power (Power = 0.151) indicates a high probability of type II error in this between‐subject effect (Figure 2).

Analysis of the interaction effect between time and condition (Time × Condition) showed significant results. The within‐subject interaction test indicated a significant interaction between time changes and type of exercise intervention (*F* (4.31, 45.33) = 66.73, *p* < 0.001). The effect size of this interaction was also reported to be very large (Partial Eta Squared = 0.864) and its statistical power was calculated to be 1.000. These results indicate that time changes in GDF‐15 levels follow different patterns depending on the type of exercise (HIIE, MICE, or control) (Figure 2).

In terms of mean descriptive changes, in the HIIE group, GDF‐15 values increased by approximately 31.1% immediately after exercise, which decreased to 21.4% by 3 h, and returned to almost baseline levels at 24 h post‐exercise (0.7% increase from pre‐test). In the MICE group, an initial increase of 13.1% was observed, which then decreased to 5.7% and then 2.2%. In the control group, GDF‐15 values did not change much, with a maximum increase of only 5.1%.

### 3.2 Acute Effects of HIIE and Continuous Moderate‐Intensity Exercise MICE on Adiponectin Levels

**FIGURE 3:**
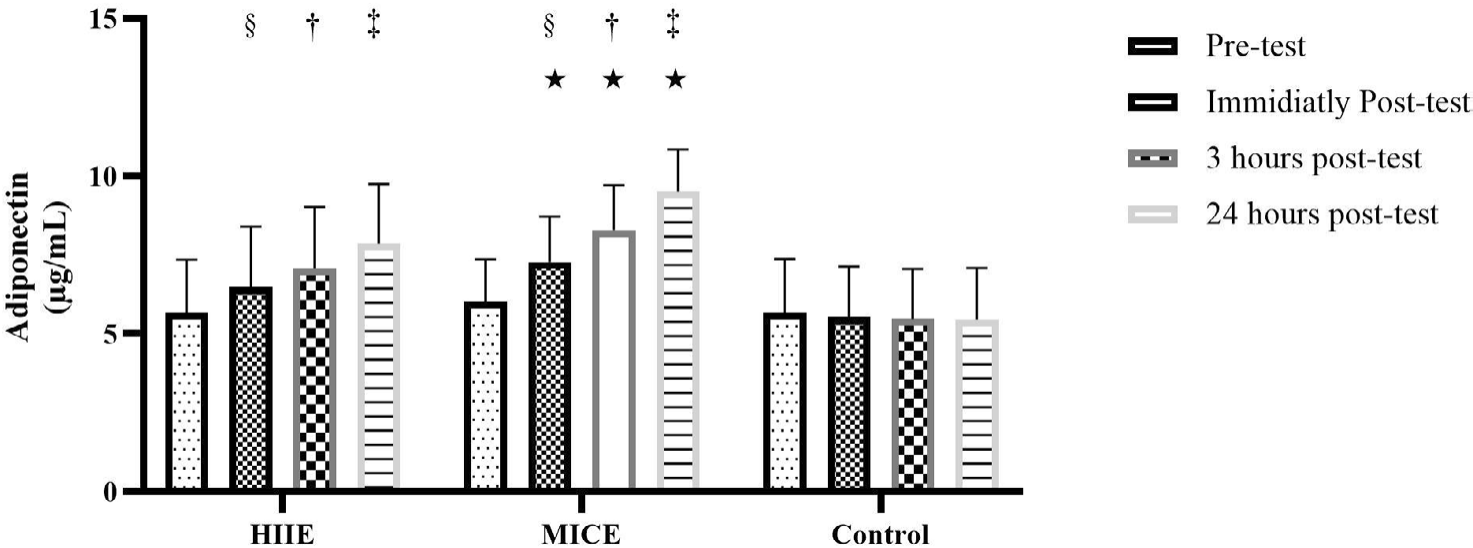
Acute effects of high‐intensity interval exercise (HIIE) and moderate‐intensity continuous exercise (MICE) on adiponectin levels. (§) Significant difference compared with pre‐test (baseline). (†) Significant difference compared with immediately post‐test. (‡) Significant difference compared with 3 h post‐test. (*) Significant difference at pre‐test, immediately post‐test, 3 h post‐test and 24 h post‐test in the control condition (*p* = 0.001).

The results of the repeated measures ANOVA showed that the effect of time on serum adiponectin levels was statistically significant. Intra‐individual analysis using the Greenhouse–Geisser correction showed that the temporal changes in adiponectin levels among subjects were highly significant (*F* (1.583, 33.246) = 185.50, *p* < 0.001). The effect size (Partial Eta Squared) for the effect of time was reported to be 0.898, indicating a very large and strong effect. Also, the statistical power (Power) for this test was 1.000, indicating complete confidence in identifying this effect (Figure 3).

Regarding the between‐group effect or the effect of training condition (Condition), the within‐subject analysis indicated that the overall mean difference in adiponectin in the three HIIE, MICE and control groups was generally significant (*F* (2, 21) = 3.850, *p* = 0.038). The effect size of this factor was reported to be 0.268 and its statistical power was 0.632, indicating a medium‐to‐high and significant effect. Paired comparison tests (Bonferroni) showed that a significant difference was observed only between the MICE and control groups (*p* = 0.034), while the difference between HIIE and other groups was not significant (Figure 3).

Importantly, the interaction effect between time and exercise condition (Time × Condition) was also statistically significant. A within‐subjects test with Greenhouse–Geisser correction showed that this interaction was *F* (3.166, 66.49) = 66.70, *p* < 0.001, with a very large effect size (Partial Eta Squared = 0.864) and a statistical power of 1.000. These results indicate that the pattern of temporal changes in adiponectin differed significantly depending on the type of exercise (Figure 3).

### 3.3 Acute Effects of HIIE and Continuous Moderate‐Intensity Exercise MICE on Leptin Levels

**FIGURE 4:**
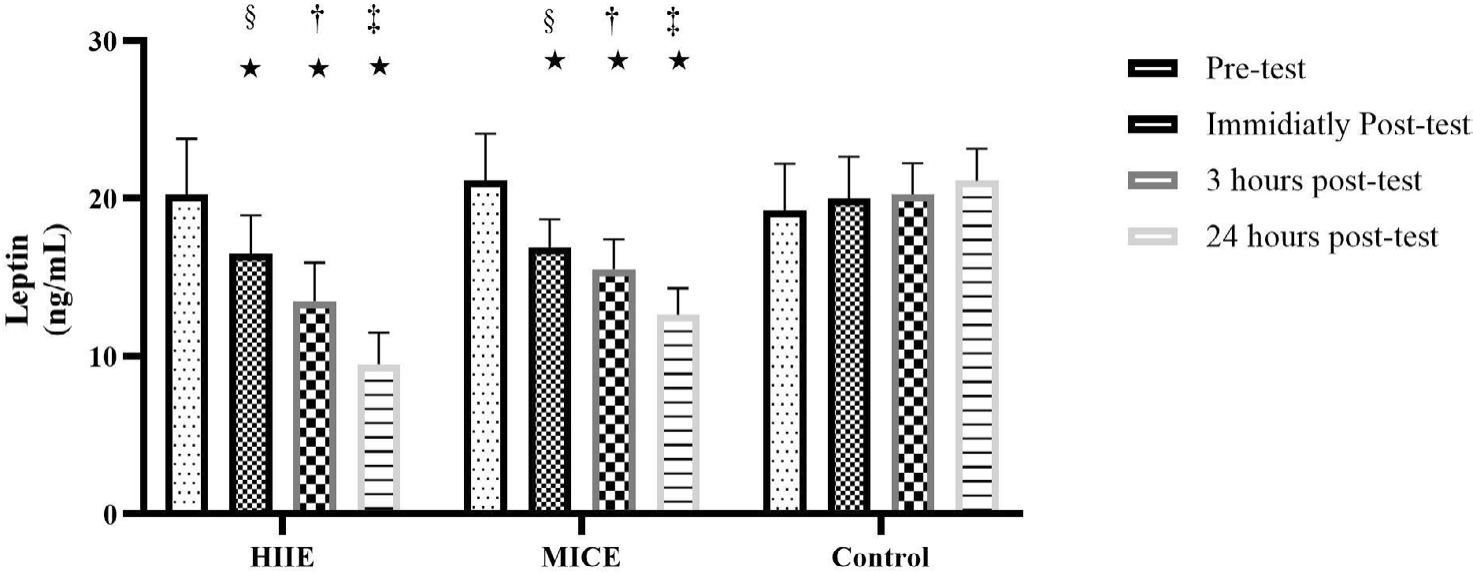
Acute effects of high‐intensity interval exercise (HIIE) and moderate‐intensity continuous exercise (MICE) on leptin levels. (§) Significant difference compared with pre‐test (baseline). (†) Significant difference compared with immediately post‐test. (‡) Significant difference compared with 3 h post‐test. (*) Significant difference at pre‐test, immediately post‐test, 3 h post‐test and 24 h post‐test in the control condition (*p* = 0.001).

The results of the statistical analysis of leptin levels in obese men with prediabetes showed that the effect of time, the effect of condition (intervention conditions) and the interaction between time and group all significantly affected leptin levels (*p* < 0.001). The time effect analysis showed that leptin levels in all participants decreased from an initial mean of 20.21 ng/mL to 18.13, 16.42 and 14.42 at immediate, 3 and 24 h after the intervention, respectively. This decrease was significant at all time points compared to baseline (*p* < 0.001), with a very strong effect size (Eta^2^ = 0.794) and full statistical power (Power = 1.000). Also, the condition effect was significant (*p* < 0.001), such that leptin levels in the HIIE, MICE and control groups were 14.94, 16.78 and 20.16 ng/mL, respectively. Pairwise comparisons indicated that the mean difference between HIIE and control (−5.22) and between MICE and control (−3.38) was significant (*p* = 0.000 and *p* = 0.014, respectively), while the difference between HIIE and MICE was not significant (*p* = 0.300). Also, the interaction of time and group also had a significant effect on leptin levels (*p* < 0.001, Eta^2^ = 0.773, Power = 1.000), such that the trend of changes in different groups showed a significant difference. In the HIIE group, leptin levels decreased from an initial value of 20.25 to 9.50 at 24 h (a 53.1% decrease). In the MICE group, a decrease was also observed from 21.13 to 12.63 (a 40.2% decrease). However, in the control group, leptin levels not only did not decrease, but also increased to 21.13 over time (a 9.8% increase). These findings suggest that both types of exercise interventions, especially HIIE, play an effective role in reducing leptin levels and improving the metabolic status of obese individuals with prediabetes, (Figure 4).

### 3.4 OGTT Response to Different Times and Interventions (HIIE and MICE)

#### 3.4.1 The Effect of Time on Glucose Levels

**FIGURE 5:**
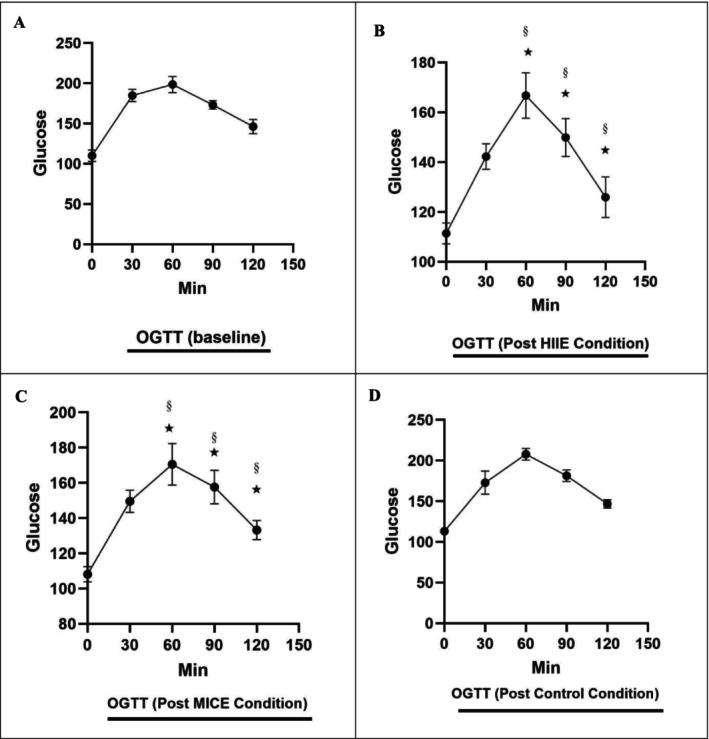
Acute effects of high‐intensity interval exercise (HIIE) and moderate‐intensity continuous exercise (MICE) on oral glucose tolerance test (OGTT). (A) Pre‐test (baseline), (B) HIIE, (C) MICE, (D) Control. (§) Significant difference compared with pre‐test (baseline). (*) Significant difference at 60, 90 and 120 min post‐test in the control condition (*p* = 0.001).

Repeated Measures ANOVA results showed that the effect of time on glucose levels during the oral glucose tolerance test (OGTT) was significant (*p* < 0.001, *F* (5, 17) = 160.757, Observed Power = 1.000). The mean glucose levels changed from 110 mg/dL at baseline (OGTTpre) to 106.75 at time zero (OGTT0), 154.88 at 30 min (OGTT30post), 181.71 at 60 min (OGTT60post), 217.08 at 90 min (OGTT90post) and 135.29 at 120 min (OGTT120post). The percent change in mean glucose from baseline was −2.95% (zero), +40.80% (30 min), +65.19% (60 min), +97.35% (90 min) and + 22.99% (120 min), respectively. Pairwise comparisons with Bonferroni adjustment showed that there were significant differences between baseline and 30, 60 and 120 min (*p* < 0.001), but the difference between baseline and 90 min was not significant (*p* = 0.908). These results indicate significant changes in glucose levels in response to the OGTT (Figure 5).

#### 3.4.2 Effect of Condition on Glucose Levels

The effect of group (HIIE, MICE and control) on the overall mean glucose levels showed that the difference between the groups was not statistically significant (*F* (2, 21) = 1.241, *p* = 0.309), (Observed Power = 0.240). The mean glucose levels in the HIIE group were 132.27 mg/dL, in the MICE group 165.25 mg/dL and in the control group 155.33 mg/dL. Pairwise comparisons with Bonferroni adjustment showed that there were no significant differences between the HIIE and MICE groups (*p* = 0.419), HIIE and control (*p* = 0.886), or MICE and control (*p* = 1.000). These findings indicate that the type of acute exercise (HIIE or MICE) had a significant effect compared to the control group. There was no significant effect on the overall mean glucose levels (Figure 5).

#### 3.4.3 The Interaction Effect of Time × Group

Analysis of the interaction effect of time and group showed that the interaction between time and group was significant (*p* = 0.001, *F* (10, 34) = 4.156, Partial Eta Squared = 0.550, Observed Power = 0.989). This result suggests that the changes in glucose levels over time are affected differently by the type of intervention HIIE, MICE or control. Specifically, at 90 min, the MICE group showed a significantly higher mean glucose (320.13 mg/dL) than the HIIE (149.88 mg/dL) and control (181.25 mg/dL) groups, indicating a high standard deviation (454.99) in this group. In the HIIE group, the percentage changes in glucose from baseline at different times were −10.11% (0), +29.32% (0.001) and −10.11% (0.001). (30 min), +51.59% (60 min), +36.25% (90 min) and +14.43% (120 min). In the MICE group, these changes were −1.70% (0), −35.91% (30 min), +55% (60 min), +191.02% (90 min) and +21.14% (120 min), respectively. In the control group, the changes were +2.95% (0), +57.16% (30 min), +88.98% (60 min), +64.77% (90 min) and +33.41% (120 min), respectively. These differences indicate different patterns in the glucose response to acute exercise compared to the control group, especially at 90 min when the MICE group showed a higher glucose peak (Figure 5A–D).

## 4 Discussion

**FIGURE 6:**
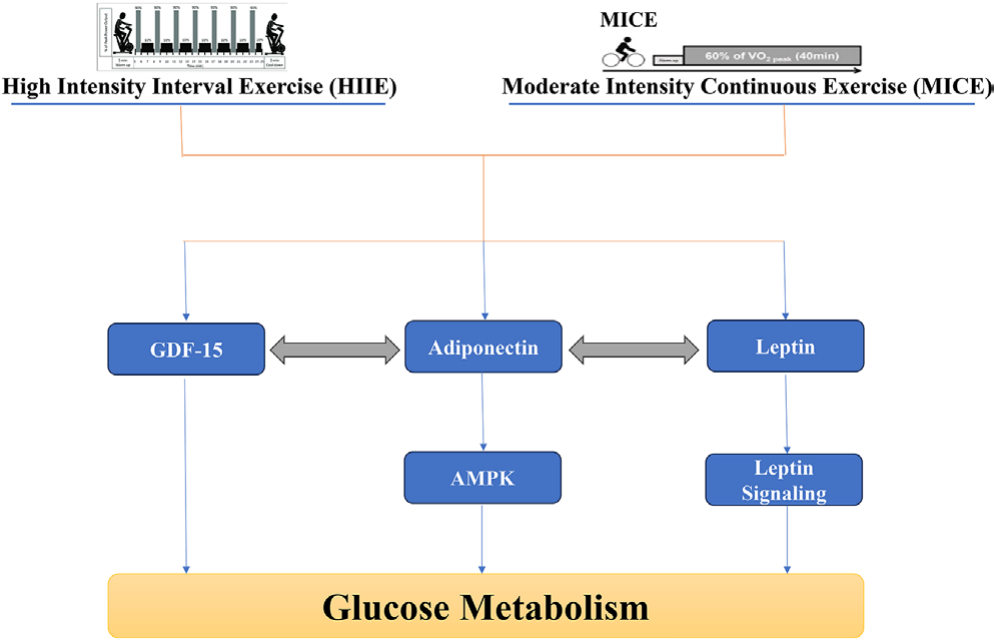
Schematic representation of the roles of GDF‐15, adiponectin and leptin in regulating glucose metabolism through AMPK‐related signalling pathways in response to high‐intensity interval exercise (HIIE) and moderate‐intensity continuous exercise (MICE).

The present study aimed to investigate the acute effects of HIIE and MICE exercise on the levels of some adipokines and their relationship with the regulation of glucose metabolism in obese men with prediabetes. The results of this study showed that acute exercise activities including HIIE and MICE exercise have different effects on serum levels of GDF‐15, adiponectin, leptin and glucose response in the oral glucose tolerance test (OGTT). The effect of time on the levels of GDF‐15, adiponectin and leptin was significant (with large Partial Eta Squared effect sizes of 0.890, 0.898 and 0.611, respectively). The interaction effect of time and the type of intervention—HIIE, MICE and control—was also significant for all three biomarkers, indicating different patterns in the responses of these markers to the type of exercise. In the HIIE group, GDF‐15 increased by 1.31% immediately after exercise, while in the MICE group it increased by 1.13% and in the control group there was little change (1.5%), (Figure 6). For adiponectin, the between‐group difference was significant, with a significant difference between MICE and control (Figure 6). Leptin levels were also affected by exercise type, with significant differences between the control and both exercise groups (Figure 6). In the case of glucose, the time effect was significant, but the between‐group effect was not significant. However, the interaction effect of time and group for glucose was also significant, with a higher glucose peak in the MICE group at 90 min.

The results of this study are consistent with some previous studies, but some differences are also observed. For GDF‐15, a study by Kleinert et al. (2018) showed that acute high‐intensity exercise caused a significant increase in GDF‐15 compared to moderate‐intensity exercise, which is consistent with the 31.1% increase in the HIIE group of this study [10].

However, the study by Zhang et al. (2020) reported that moderate‐intensity continuous exercise can also produce sustained increases in GDF‐15, which is somewhat consistent with the 13.1% increase in the MICE group of this study, but the lower persistence may be due to differences in exercise intensity or duration [17]. The heterogeneity in the between‐group effect may be due to the small sample size or low statistical power (0.151), which increases the likelihood of a type II error.

The acute increase in GDF‐15 in response to HIIE training may be due to the more severe metabolic stress caused by the high‐intensity intermittent bouts. GDF‐15 is known as a metabolic stress marker that is released in response to tissue damage or inflammation [22]. In HIIE, high energy demand and accumulation of metabolites such as lactate can lead to activation of GDF‐15‐related signalling pathways, including the AMPK pathway [23]. However, the magnitude of this response may vary depending on the individual's metabolic status. In prediabetic individuals, who have lower baseline levels of systemic inflammation and maintain greater metabolic flexibility compared with obese or type 2 diabetic patients, the GDF‐15 response to acute exercise may be more pronounced. In contrast, in overt diabetes or long‐standing obesity, high levels of GDF‐15 and disrupted signalling pathways may blunt the acute response of this adipokine to exercise stimuli.

For adiponectin, the results of this study are consistent with the findings of Saunders et al. (2016), who showed that moderate‐intensity continuous exercise significantly increased adiponectin compared to the control group [24]. However, the lack of a significant difference between HIIE and control in this study is in contrast to the results of Tjønna et al. (2008) who reported that HIIE can induce stronger adiponectin responses; in this study no significant difference was observed between HIIE and control. This discrepancy may be due to differences in training protocols (such as duration or intensity of HIIE sessions) or characteristics of the study population (such as age, BMI or fitness level) [25]. On the other hand, the increase in adiponectin in the MICE group is also associated with the activation of the same AMPK pathway as well as the PPARα pathway. Adiponectin activates metabolic pathways related to fatty acid oxidation and glucose uptake through the AdipoR1 and AdipoR2 receptors, which play a role in improving insulin sensitivity [26, 27]. Moderate‐intensity continuous exercise (MICE) appears to have a greater effect on increasing adiponectin than HIIE due to more sustained stimulation of these pathways (Figure 2).

On the other hand, leptin, a hormone associated with body fat and energy balance, decreased under the influence of both types of training. In the case of leptin, the results of this study were consistent with the study by Rodrigues et al. (2020), with results showing a significant effect of time (*F* = 17.52, *p* = 0.006) with no difference between training conditions or interaction. Leptin decreased similarly in both conditions and was not significantly different between HIIE and MICE. This pattern is in good agreement with the strong effect of time and the lack of difference between the two training conditions [28]. However, studies by Sim et al. (2014) and Larsen et al. (2018) reported that HIIE had a stronger effect on leptin reduction, which is inconsistent with the lack of significant difference between HIIE and MICE in this study [29, 30].

Oniz et al. (2023) showed in their study that leptin levels decreased significantly over time after 2 weeks of HIIT training, but there was no difference between groups (between training and control). This is also different from the interpersonal effect where the type of training is important [31]. This difference may be due to the shorter follow‐up period in this study or the difference in exercise intensity and subject type, as in the present study, obese men were prediabetic, while in the study by Sim and et al., the subjects were overweight.

Reduced leptin may be a result of reduced adipose tissue as well as reduced inflammation and oxidative stress [32], As it has been reported that adiponectin‐leptin plays an important role in inflammation and oxidative stress [32]. Therefore, it can be said that the increase in adiponectin and decrease in leptin induced by both HIIE and MICE exercise probably has a positive effect on glucose metabolism through the reduction of inflammation and oxidative stress. Interestingly, the AMPK pathway, previously identified as a common pathway of GDF‐15 and adiponectin, plays an important role in the suppression of leptin signaling and its negative regulation [33, 34]. Therefore, it is possible that the interaction between these three indicators occurs through the co‐regulation of the AMPK pathway and the response to metabolic stress induced by different exercise training. In addition, differences in glucose response may also complement the understanding of this relationship. While HIIE leads to faster glucose uptake through non‐insulin‐dependent pathways such as increased GLUT4 activity, MICE is more dependent on insulin‐dependent pathways [35]. This difference is also linked to the levels of adipokines, particularly adiponectin and leptin, which are involved in regulating insulin sensitivity [36], (Figure 6).

Regarding the results related to glucose levels, the findings of this study are consistent with the findings of the meta‐analysis of Khalafi et al. (2022) which showed that HIIE exercises can improve glucose response [37], but the lack of between‐group differences is inconsistent with the findings of Little et al. (2011) who reported that HIIE has a greater effect on glucose regulation [38]. This discrepancy may be due to the high standard deviation in the MICE group at 90 min or differences in study design.

Among the limitations of this study was the relatively small sample size (*n* = 8), which may have reduced the statistical power of the analyses and resulted in the failure to detect real differences, especially in variables such as GDF‐15. The lack of control of the participants' diet in the pre‐ and post‐intervention period is another limitation that could affect adipokines levels. Also, the short‐term follow‐up (24 h after exercise) may not have been sufficient to examine sustained changes in markers such as adiponectin and leptin. Differences in individual characteristics of the participants such as BMI, age and fitness level, despite statistical control, could also affect the results. Finally, the lack of data on some important biomarkers such as insulin and IL‐6 limits the analysis of the physiological mechanisms associated with the GDF‐15 and adiponectin response.

## 5 Conclusion

This study found that acute HIIE and MICE exercise affected the biomarkers GDF‐15, adiponectin, leptin and glucose response (Figure 6). Although the patterns suggest that HIIE may be associated with a more acute increase in GDF‐15 and MICE with a more sustained effect on adiponectin, these differences were not statistically significant and should therefore be interpreted with caution. Both types of exercise were associated with a reduction in leptin, and differences in glucose response patterns were observed, particularly at 90 min. Overall, these findings suggest that the type and intensity of exercise may play a role in metabolic regulation, but further studies with larger sample sizes, dietary controls and longer follow‐up are needed to clarify these mechanisms.

## Author Contributions

H.G. and M.R.R. wrote the study protocol and reviewed the results. H.G., E.E. and M.M. performed the statistical analysis. M.R.R. reviewed the results and the manuscript. H.G., M.M. and E.E. extracted the database records and drew the figures. H.G. and M.R.R. prepared the study protocol and wrote the manuscript. All authors revised and approved the final manuscript.

## Conflicts of Interest

The authors declare no conflicts of interest.

## Supporting information


**Data S1:** edm270125‐sup‐0001‐supinfo.zip.

## Data Availability

The data supporting this study's findings are available from the corresponding author upon reasonable request.
